# A Dynamic Intrusion Detection System Based on Multivariate Hotelling's T^**2**^ Statistics Approach for Network Environments

**DOI:** 10.1155/2015/850153

**Published:** 2015-08-18

**Authors:** Aneetha Avalappampatty Sivasamy, Bose Sundan

**Affiliations:** Department of Computer Science and Engineering, College of Engineering Guindy, Anna University, Chennai 600025, India

## Abstract

The ever expanding communication requirements in today's world demand extensive and efficient network systems with equally efficient and reliable security features integrated for safe, confident, and secured communication and data transfer. Providing effective security protocols for any network environment, therefore, assumes paramount importance. Attempts are made continuously for designing more efficient and dynamic network intrusion detection models. In this work, an approach based on Hotelling's T^2^ method, a multivariate statistical analysis technique, has been employed for intrusion detection, especially in network environments. Components such as preprocessing, multivariate statistical analysis, and attack detection have been incorporated in developing the multivariate Hotelling's T^2^ statistical model and necessary profiles have been generated based on the T-square distance metrics. With a threshold range obtained using the central limit theorem, observed traffic profiles have been classified either as normal or attack types. Performance of the model, as evaluated through validation and testing using KDD Cup'99 dataset, has shown very high detection rates for all classes with low false alarm rates. Accuracy of the model presented in this work, in comparison with the existing models, has been found to be much better.

## 1. Introduction

Sophisticated security policies and tools are designed continuously in order to ensure integrity, availability, and confidentiality of data for legitimate users in a network environment. Security tools such as firewall and cryptographic techniques and authentication are designed based on the attacks existing at the time of their development [1]. However, malicious users nowadays observe and analyze communication networks continuously for possible vulnerabilities in order to gain unauthorized access to the system. Therefore, there is always a need for better detection mechanisms capable of analyzing user activities and classifying them either as legitimate or malicious ones on a real time basis [2]. Extensive work is being carried out by a large number of investigators to develop such real time intrusion detection systems (IDS) for providing complete network security [1–10].

Intrusion detection systems generally analyze and dynamically monitor network traffic patterns and log information. The analysis helps in deploying suitable detection methodologies to identify whether the events have any signature of attacks or are legitimate profiles [2]. Based on detection methodologies, the IDS architecture is categorized either as misuse detection or anomaly detection [3]. Misuse detection monitors network activities and compares the traffic profile with existing attack and normal profiles. This approach achieves higher detection and low false positive rates for existing attacks but any new attack or even small deviations from existing attacks may not get detected easily [2]. On the other hand, anomaly detection monitors the traffic by comparing the observed profile with legitimate traffic profiles and if the observed profile deviates significantly from the legitimate profile then it is signaled as an anomaly. Though this method achieves high detection rates, there are possibilities of misjudgment of legitimate profiles leading to a high false positive rate [11]. An ideal IDS system should therefore have a high detection rate while keeping the false positive rate as minimum as possible.

Attempts have been made for enhancing detection performance and efficiency of IDS systems for anomaly detection using a wide range of algorithms. These algorithms are largely based on data mining [4, 7, 10], machine learning [1, 5, 6], and statistical techniques [11–13]. Algorithms based on data mining and machine learning approaches, in general, are based on their computational intelligence and achieved good detection rates. However, very often, they result in high false positive rates because the relationships between features are not given adequate attention [14]. Detection techniques based on statistical methods use parameters such as mean, variance, and standard deviation to build the legitimate profile system. Statistical tests identify the deviation in an observed traffic profile from the legitimate ones and accept the deviation if it is within the permissible level of significance. An advantage of this approach is its ability to handle noise and variances in the system explicitly [15].

Network traffic profiles are often characterized by multiple features. Any deviations caused in such multiple attributes also need to be considered while analyzing the network for intrusions. Therefore, profiles represented by multiple attributes need to employ multivariate analysis techniques for analyzing traffic profiles. This approach can eliminate the problem of comparing a predicted event with an observed event directly [13] which would possibly reduce false alarm rates.

Hotelling's T^2^ test, a multivariate statistical technique, has been developed as a process control tool used for hypothesis testing [15–19]. This approach identifies the correlation between variables using covariance matrix based on which the process control model is constructed. Identifying the correlation assumes importance in order to find out the probability of rejecting the null hypothesis (observed profile is normal and belongs to the model) by accepting the alternative hypothesis (observed profile is an anomaly and does not belong to the model) [19]. Hotelling's T^2^ test has been employed for tracking an object in a video stream by comparing its multivariate mean in successive frames. It is reported that the test is capable of perfectly detecting both moving and stationary objects [20]. Potential problems likely to be encountered and possible solutions when using Hotelling's T^2^ technique have been discussed by Sparks for the processes in which data are highly correlated [21].

Ye et al. have carried out multivariate statistical analysis of audit trails for detecting intrusions in host systems using Hotelling's T^2^ technique and detected both counterrelationship and mean shift anomalies. For smaller datasets, all intrusions are detected with zero false alarm rates whereas, for larger datasets, the detection rate has been 92% with zero false alarm rates [15]. They have also carried out studies using Chi-square multivariate test and Markov chain process for detecting intrusions in computer audit data. Analysis of probabilistic properties such as frequency and ordering has been carried out to perform detection process. It is reported that under certain situations frequency property is found to give better detection rates with less computational overhead [12]. However, they have considered providing security mainly for host machines using audit data. An improvement reported in their work is that the model results in zero false alarm rates, a desirable feature for any efficient intrusion detection system.

### 1.1. Contributions of the Present Work

Though numerous intrusion detection systems have been developed for providing security for network environments, very often it is reported that false alarm rates need to be considerably reduced or eliminated. Since the Multivariate Hotelling's T^2^ Statistical (MHT^2^S) technique for intrusion detection in host machines has been reported to produce zero false alarm rates, it is possible to employ this approach for providing security in a dynamic network environment as well. Studies employing MHT^2^S model for anomaly detection in network environment, to our knowledge, are very rare. Therefore, in this work, a network anomaly detection system based on MHT^2^S technique is developed with an objective of achieving high detection rates combined with low false alarm rates.

The MHT^2^S model is built with legitimate traffic profiles and the statistical deviation of an observed traffic profile from the legitimate ones is measured. If the statistical deviation of an observed traffic falls outside the specified threshold range, the observed traffic is then suspected as an anomalous one. The threshold range is calculated using the central limit theorem for multivariate analysis. The performance of the anomaly detection system proposed in this work is evaluated using the benchmark KDD Cup'99 dataset.

The paper is organized as follows: Section 2 gives the description of KDD Cup'99 dataset and preprocessing and describes Hotelling's T^2^ statistical methodology with its attack detection mechanism in detail.  Section 3 presents the results of the present work and discusses the performance of the proposed model. The results are analyzed and compared with the existing anomaly detection techniques.  Section 4 gives the important conclusions of the MHT^2^S model and its performance.

## 2. Data Source and Methodology

### 2.1. Dataset Description

The KDD Cup'99 dataset [22], the most widely used and accepted benchmark dataset for network intrusion detection systems, has been used to evaluate the performance of the proposed MHT^2^S methodology. Though it is criticized for redundancy, the labeled profiles of this dataset serve as an effective source for comparing the performance of any new intrusion detection approach with other approaches. The 10%-corrected–subset-KDD Cup'99 dataset is used in this work. It has 21 different types of attacks along with normal profile. The attacks are broadly divided into 4 types, namely, denial of services (DoS), unauthorized access to local supervisor privileges (U2R), unauthorized access from a remote machine (R2L), and surveillance and other probing (probe). Each network profile is represented by 41 different features along with the class feature. Among the 41 features, 32 are continuous, 3 are categorical, and 6 are nominal features. The complete description of all the features is available in literatures [1, 10].

### 2.2. Methodology

#### 2.2.1. Preprocessing

The KDD Cup'99 dataset is collected from a simulated environment and information available needs to be processed before it is used for developing any intrusion detection system. Four steps of preprocessing have been carried out for the dataset in order to make them suitable for developing the MHT^2^S model. They are redundancy removal, numeric value assignment, normalization, and feature selection. In the preprocessing step, eliminating redundant traffic profiles of the data source makes the model unbiased towards any recurring traffic profile.  Table 1 presents the number of samples of the dataset before and after eliminating redundant records and its percentage contributions. In the second step, categorical features such as protocol type, flag, and services are assigned with numeric values to perform statistical calculations. For example, protocol type features have three possible values, namely, TCP, UDP, and ICMP, and are assigned with numeric values 1, 2, and 3, respectively.

After assigning numeric values, the range of values for different features is different.  Table 2 shows the details of some of the features and their maximum and minimum and number of distinct values. Therefore, a suitable normalization technique becomes necessary for developing the MHT^2^S system to avoid domination of features with wider range over the ones with narrow range. In this work, Min-Max normalization technique [23] has been employed to linearly scale the range of feature values from 0 to 1 for all features using

(1) $$\begin{array}{c} F_{\left(\text{MM}\right)j}=\frac{F_{j\left(\text{old}\right)}-F_{j\left(\min\right)}}{F_{j\left(\max\right)}-F_{j\left(\min\right)}}, \end{array}$$

where *F*_*j*(old)_ denotes original value, *F*_(MM)*j*_ is the new scaled value, and *F*_*j*(min⁡)_ and *F*_*j*(max⁡)_ represent the minimum and maximum values of *j*th attribute, respectively. After normalization, features are analyzed for their significance towards the MHT^2^S intrusion detection model. For example, correlation between features could influence the results due to the possible elimination of features randomly which is likely to decrease the accuracy. Some features might have no effect at all or contain a high level of noise and therefore their removal can increase the speed and accuracy rate of the system [10]. Therefore threshold based feature selection is carried out here.

#### 2.2.2. Multivariate Hotelling's T^2^ Statistics

T-square distance (TSD) method is used in statistics for hypothesis testing of both univariate and multivariate applications. This technique can identify whether an observed profile belongs to a particular group or not. This technique utilizes first order statistical measures such as mean and variance along with second order statistical measures such as sample covariance matrix for hypothesis testing. These statistical measures analyze correlations between variables and remove dependencies on the scale of measurement during calculation [14]. In this work, TSD method is used to measure the difference between legitimate traffic profiles and observed traffic profiles for anomaly detection.

Consider a set of *N* legitimate training profiles represented as *X* ^Normal^ = {*x*_1_^normal^, *x*_2_^normal^, *x*_3_^normal^,…, *x*_*N*_^normal^}, where each profile is represented as *x*_*i*_^normal^ = {*x*_*i*1_^normal^, *x*_*i*2_^normal^, *x*_*i*3_^normal^,…,*x*_*iP*_^normal^}^T^ with *P* feature vectors. After preprocessing and normalization, each original legitimate traffic profile *x*_*i*_^normal^ is transformed into *x*_MM_*i*__^normal^ which is denoted as *x*_MM_*i*__^normal^ = (*x*_MM_*i*1__^normal^, *x*_MM_*i*2__^normal^, *x*_MM_*i*3__^normal^,…,*x*_MM_*iP*__^normal^)^T^. The TSD is computed for every preprocessed training traffic profile using

(2) TSDiNormal=N∗X−MMNormal−XMMiNormalT∗SP×PNormal−1∗X−MMNormal−XMMiNormal,

where ${\overset{-}{X}}_{\text{MM}}^{\text{Normal}}\;$is the mean feature vector and *S*_*P*×*P*_^Normal^−1^^ is the inverse sample covariance matrix of size *P* × *P* as represented in

(3) $$\begin{array}{c} S_{P\times P}^{\text{Normal}}=\left[\begin{array}{ccc} S_{11} & S_{12} & S_{1P}\\ \vdots & \vdots & \vdots \\ S_{P1} & S_{P2} & S_{PP} \end{array}\right], \end{array}$$

where *S*_*i*,*j*_ is the covariance between features *i* and *j* and is calculated using (4) or (5). Consider the following:

(4) Sij=1N−1∑k=1NXMMi,kNormal−X−MMiNormalXMMj,kNormal−X−MMjNormalif  i≠j,

(5) Sij=1N−1∑k=1NXMMi,kNormal−X−MMiNormal2if  i=j.

Various steps in developing MHT^2^S model along with the generation of normal profiles based on TSD are given in Algorithm 1. TSD values of each profile are used for calculating mean (*μ*_TSD_) and standard deviation (*σ*_TSD_) as given in the algorithm. These values are employed in calculating the threshold range using central limit theorem. The calculated threshold range is used in the attack detection module.

**Algorithm 1 (MHT^2^S).**

Consider the following.

*Input*. *X*_MM_^Normal^ = {*x*_MM1_^normal^, *x*_MM2_^normal^, *x*_MM3_^normal^,…, *x*_MM*N*_^normal^} each with *P* feature vector.

*Output*. TSD for each traffic profile, *S*_*P*×*P*_^Normal^, *μ*_TSD_, *σ*_TSD_.

(1) Calculate sample mean ${\overset{-}{X}}_{\text{MM}}^{\text{Normal}}=\;\left(1/N\right)\sum_{i=1}^{N}x_{{MM}_{i}}$ for *P* vector.
(2) Generate sample covariance matrix *S*_*P*×*P*_^Normal^ using (3).
(3) For *i* = 1 to *N* do
   calculate ${TSD}_{i}=TSD(X_{MM}^{Normal},{\overset{-}{X}}_{MM}^{Normal})$
     {T-Squared Distance between *X*_MM_*i*__ and ${\overset{-}{X}}_{MM}$ computed using (2)}
 End for
(4) Compute mean of TSD *μ*_TSD_ ← (1/N)∑_*i*=1_^*N*^TSD_*i*_
(5) Compute standard deviation $\sigma_{TSD}=\sqrt{\left(1/\left(N-1\right)\right)\sum_{i=1}^{N}{\left({TSD}_{i}-\;\mu_{TSD}\right)}^{2}}$
(6) Return $\left(\mu_{TSD},\sigma_{TSD},S_{P\times P}^{Normal},{\overset{-}{X}}_{MM}^{Normal}\right)$.

#### 2.2.3. Attack Detection

TSD value is calculated for the observed traffic profile using sample mean vector and sample covariance matrix. TSD value thus obtained is transformed into T^2^ statistic by multiplying TSD with a constant value as given in (6), which follows *F* distribution. Consider

(6) $$\begin{array}{c} \frac{N\left(N-P\right)}{P\left(N+1\right)\left(N-1\right)}\text{TSD}, \end{array}$$

where *N* is the number of sample traffic profiles and *P* is the number of feature vectors. If the transformed T^2^ statistic is greater than the corresponding *F* table value, the observed profile is then signaled as an anomaly [19]. Since network traffic profiles have multiple features and when samples are more than 30, the above transformation method and *F* table values need not be used as threshold for anomaly detection [15].

Instead, central limit theorem is used for detecting multivariate network traffic samples with the assumption that TSD value of multivariate profiles approximately follows normal distribution. Taking TSD values as samples, the mean and standard deviations are calculated for estimating the threshold range. The threshold range is given by *μ*_TSD_ + *λ∗σ*_TSD_ and *μ*_TSD_ − *λ∗σ*_TSD_ as upper and lower limits, respectively. *μ*_TSD_ is the mean and *σ*_TSD_ is the standard deviation of the TSD values of normal profiles. *λ* is the level of confidence, usually ranging from 1 to 3 for confidence levels ranging from 68% to 99.7% [14]. The observed traffic profiles are preprocessed and represented as *x*_MM1_^observed^ = (*x*_MM11_^observed^, *x*_MM12_^observed^, *x*_MM13_^observed^,…,*x*_MM1*P*_^observed^)^T^. TSD^observed^ value is calculated for the observed traffic profile using sample mean vector and covariance matrix of the normal traffic profiles. If the TSD^observed^ value is out of the threshold range, then it is signaled as an attack. The formal flow of the detection mechanism is given in Algorithm 2.

**Algorithm 2 (attack detection).**

Consider the following.

*Input*. *X* ^Observed^, *μ*_TSD_, *σ*_TSD_, *S*_*P*×*P*_^Normal^, ${\overset{-}{X}}_{MM}^{Normal}$, *λ*.

*Output*. normal or attack

(1) Generate *X*_MM_^Observed^ after performing pre-processing
(2) ${TSD}^{Observed}\leftarrow TSD\left(X_{MM}^{Observed}-{\overset{-}{X}}_{MM}\right)$ using
   
${TSD}^{Observed}=N\cdot {\left({\overset{-}{X}}_{\text{MM}}^{\text{Normal}}-X_{MM}^{Observed}\right)}^{T}\cdot S_{P\times P}^{{Normal}^{-1}}\cdot ({\overset{-}{X}}_{MM}^{Normal}-X_{MM}^{Observed})$
(3) If (*μ*_TSD_ − *σ*_TSD_ *∗λ*) ≤ TSD^Observed^ ≤ (*μ*_TSD_ + *σ*_TSD_ *∗λ*) then
(4) Return normal
(5) Else
(6) Return attack.
(7) End if.

## 3. Results and Discussions

### 3.1. Evaluation Metrics

The MHT^2^S intrusion detection system has been evaluated in terms of system accuracy, attack detection rate, and false alarm rate. Accuracy (acc) of a complete system is the ratio of the sum of normal and abnormal records correctly identified to the total number of records using

(7) $$\begin{array}{c} \text{acc}=\frac{\sum_{i=1}^{c}\left[{\text{TP}}_{i}+{\text{TN}}_{i}\right]}{N}, \end{array}$$

where *c* is the number of classes and *N* is the total number of records. Detection rate (DR) is given as the ratio of the number of correctly classified records in a particular class to the total number of records of that class and is given by

(8) $$\begin{array}{c} \text{DR}=\frac{\text{TP}}{\left(\text{TP}+\text{FP}\right)}. \end{array}$$

False alarm rate (FAR), also referred to as false positive rate, is the ratio of the number of incorrectly generated alarms for normal records to the total number of normal records [2] given by

(9) $$\begin{array}{c} \text{FAR}=\frac{\text{FP}}{\left(\text{FP}+\text{TN}\right)}, \end{array}$$

where TP, TN, FP, and FN are true positive, true negative, false positive, and false negative, respectively. TN is attacks correctly detected as attacks; TP is normal correctly classified as normal; FP is normal incorrectly classified as attack; and FN is attack incorrectly classified as normal.

Apart from these metrics, the visualization tool used for analyzing the performance of the intrusion detection system is the Receiver Operating Characteristic (ROC) curve. The ROC curve provides a clear trade-off between detection rate and false alarm rate for every model. Values that appear in the upper left triangle of the ROC curve, that is, above the line *y* = *x*, are a clear indication of good performance of a classification model [12, 15, 19].

### 3.2. Experimental Description

The proposed MHT^2^S intrusion detection model was developed on a personal computer with the processor Intel(R) Core i5 – 2410 M, CPU @ 2.30 GHz, 5 GB of memory, and 32-bit Windows 7 Ultimate operating system. The algorithm was implemented in NetBeans IDE 7.0 platform with JAVA SE7 version. The MHT^2^S intrusion detection model has been evaluated using the KDD Cup'99 dataset. The MHT^2^S based DoS model utilized 54574 unique DoS profiles. Out of these profiles, 50574 were used for building the model and the remaining 4000 profiles for testing the model. In the probe model, out of 1628 unique profiles, 1478 were used for building and the remaining 150 for testing the model. In the R2L model, 375 unique profiles were used for building the model and remaining 50 for testing. In the U2R model, 32 profiles were used for building the model and 5 for testing the model. In case of normal model, 50000 unique profiles were selected proportionately from 87832 profiles. Out of the 50000 selected profiles, 45000 were used for building the model and the remaining 5000 were used for testing the MHT^2^S based normal model. The number of features selected after preprocessing in DoS, probe, R2L, U2R, and normal models is 13, 23, 13, 20, and 15, respectively, and the names of the features are listed in Table 3.

### 3.3. Results

The results obtained are discussed in this section. In this study, separate detection models are developed for normal and four types of attacks based on their history of unique traffic profiles available in the KDD Cup 10% subset of the corrected traffic profiles. Each model is evaluated first by validation followed by testing process. While validation is performed to measure the generalized capacity of the system with the same traffic profile, testing is performed in order to define the efficiency of the proposed IDS with same and attack traffic profiles.

#### 3.3.1. Tenfold Cross Validation

Validation of MHT^2^S detection system has been carried out using tenfold cross validation technique. The advantage of this technique is that it gives a reduction in variance which makes the results of the model less sensitive towards different training groups. In tenfold cross validation process, legitimate traffic profiles are divided into ten sets from which a training dataset is created by combining randomly selected nine sets to build the MHT^2^S detection system. The remaining is used as test dataset for evaluating the performance of the model. The process is repeated ten times by combining datasets in ten different ways and the average detection rate is considered as the result of the system. For example, results obtained using tenfold cross validation of the DoS model are shown in Table 4. The performance of the systems is studied using *λ* values ranging from 1 to 3 for determining the threshold range.

The average detection rates thus obtained in tenfold validation for all the models with different threshold ranges are given in Table 5. It is observed from Table 5 that, throughout the validation process, the model has been able to achieve better performance for all the classes as the *λ* value increases from 1 to 3. Due to an increase in the threshold bandwidth, the detection rate of the normal model has been found to be at maximum with a *λ* value of 3 as the level of confidence increases. In the case of DoS model, the change in detection rate is relatively less as a function of *λ*. This drop in detection rate could be attributed to a relatively less number of DoS profiles when compared to the normal profiles. For probe and R2L profiles, as the threshold range increases, the detection rates have increased significantly from 91.55 to 98.07 percent and 89.50 to 98.25, respectively. However, the system is found to achieve only 60 percent detection rate in case of U2R model even with larger threshold bandwidth. This could be due to the fact that the number of available traffic samples is much less, with only 32 profiles for training and 5 profiles for testing the model.

#### 3.3.2. Performance Testing

Performance testing of MHT^2^S detection system has been carried out using the training dataset consisting of 90% of normal traffic profile. Remaining 10% of normal profile has been combined with 10% of attack profiles to form the test dataset. For example, out of 54572 unique DoS traffic records, 50572 records are taken as training dataset and used for developing the MHT^2^S DoS model and the remaining 4000 records are combined with equal number of normal records as test dataset.

**Figure 1:**
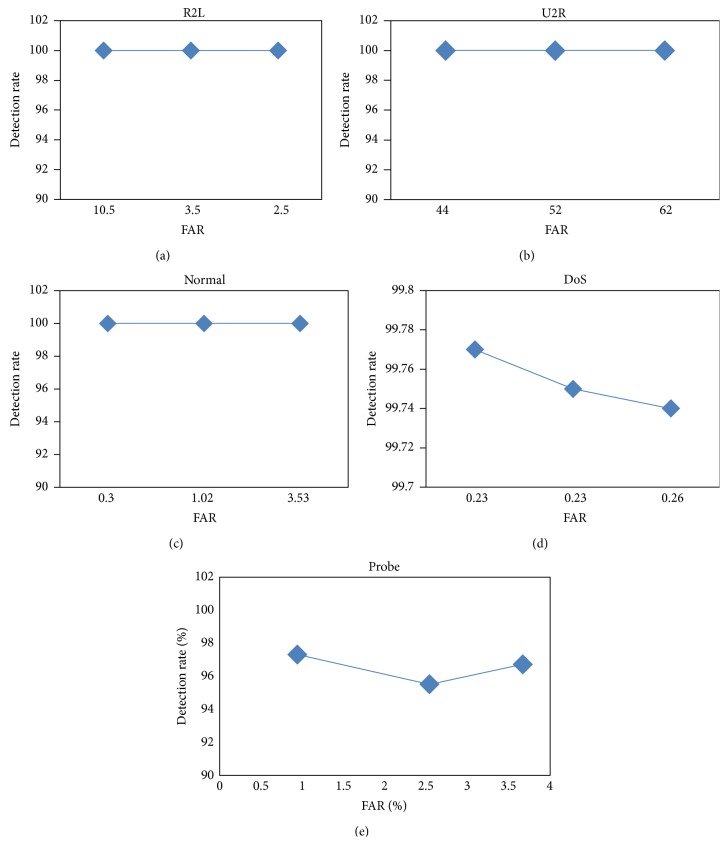
ROC curve for all classes.

During the evaluation process, both training and test datasets are kept entirely different in such a way that the model provides a more generalized environment for predicting its efficiency. The performance testing has been carried out by keeping the *λ* values at 1, 2, and 3. The results obtained for smaller variations in *λ* values, say, 1 and 1.5, are more or less the same. The results of the performance tests are plotted as ROC curves in Figure 1. The results reveal that the MHT^2^S model has been able to achieve 100% detection rate for normal, R2L, and U2R classes whereas, for the DoS and probe classes, the model achieves 99.77 and 97.32 percent detection rates, respectively. The false alarm rates obtained using this model for normal, R2L, U2R, DoS, and probe classes are 0.30, 2.50, 44, 0.23, and 0.94, respectively, which are shown in Table 6.

The detection system has been found to be efficient based on the ROC curves which provide a good trade-off between detection rates and false alarm rates for all the classes. Figure 1 clearly shows that, for all the classes, results occupy the upper left triangle of the graph. This is a good indication of an efficient classification model based on the concept of ROC curve.  Table 7 shows the accuracy of MHT^2^S detection model for normal, DoS, probe, R2L, and U2R classes for different threshold ranges with *λ* values 1, 2, and 3. The accuracy rates achieved for normal and DoS models are more than 99% and do not vary significantly with respect to changes in the threshold. For the probe class, however, the accuracy drops significantly from 96.88 to 59.31 as the *λ* value increases from 1 to 3. For R2L and U2R classes, the accuracy rates are found to be at maximum for a *λ* value of 2.

**Figure 2:**
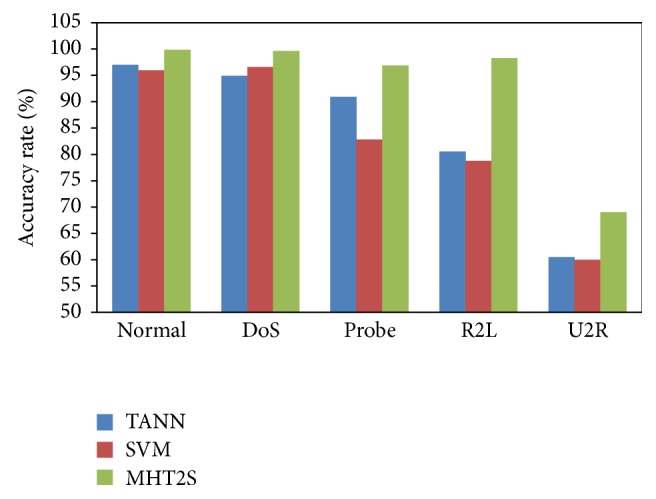
Performance comparison.

Performance of MHT^2^S model in terms of detection rate, false alarm rate, and accuracy for all classes is found to be better than the results obtained with the best detection approaches published. Accuracy of MHT^2^S model is compared with the results in the literature [4] and it is shown in Figure 2. The MHT^2^S approach is capable of analyzing each feature based on statistical parameters and their relationships. Therefore, any small deviations in the features would not have any significant impact on their relationship and hence the results do not change significantly. This is an advantage of the MHT^2^S model for network intrusion detection.

## 4. Conclusions

A new approach for intrusion detection in network environments has been presented by deploying Hotelling's T^2^ statistical test, a multivariate process control technique. The MHT^2^S detection system is developed in three steps, namely, preprocessing, multivariate Hotelling's T^2^ statistics, and attack detection. Redundancy removal, normalization, and selecting relevant features are carried out in preprocessing step. Using Hotelling's T^2^ statistics, profiles are generated based on T-square distance metrics. Attack detection is implemented by determining a threshold range using central limit theorem. Based on the determined threshold range observed profiles are classified either as normal or attack. The MHT^2^S model is evaluated using KDD Cup'99 dataset to verify its effectiveness.

Performance of the model has been evaluated through validation and testing. Validation has been performed for analyzing the model for its detection rate based on traffic profiles. Testing helped in understanding the significance of the model through unknown and known attack profiles for each class. The results have shown encouraging performance in terms of detection rate and false alarm rate. 100 percent detection rates are achieved for normal, R2L, and U2R classes. For DoS and probe classes the detection rates are at 99.77 and 97.32 percent, respectively. Very low false alarm rates are achieved for all classes except U2R. For U2R, the false alarm rate is found to be considerably high due to the less number of traffic profiles. Comparing the accuracy of the model presented in this work with the existing models, it is found that the MHT^2^S based intrusion detection model achieves better performance. Therefore, MHT^2^S model could be employed as an effective tool for providing security for network environments. A better mechanism needs to be designed to reduce false alarm rate for the U2R class which could be explored in the future.

## Figures and Tables

**Table 1:** Description of redundancy in dataset (10%_corrected_subset_KDD Cup'99).

Class	Number of original records		Number of records after redundancy removal	
	Number of samples	%	Number of samples	%
Normal	97279	19.75	87832	60.79
DoS	391460	79.46	54573	37.77
Probe	3460	0.70	1627	1.13
R2L	442	0.08	425	0.29
U2R	37	0.01	37	0.03
Total	492678	100	144494	100

**Table 2:** Minimum, maximum, and distinct values of some features of KDD Cup'99.

Features	Min	Max	Distinct
Protocol type	1	3	3
Flag	1	11	11
Service	1	66	66
src_bytes	0	693375640	3300
dst_bytes	0	5155468	10725
diff_srv_rate	0	1	78
dst_host_same_src_port_rate	0	1	101
Count	0	511	490

**Table 3:** Features selected for building MHT^2^S model.

Class	Selected features
DoS	Protocol type, service, flag, src_bytes, dst_bytes, count, srv_count, serror_rate, srv_serror_rate, dst_host_count, dst_host_srv_count, dst_host_serror_rate, dst_host_srv_serror_rate.

Probe	Duration, protocol_type, service, flag, src_bytes, dst_bytes, count, srv_count, srv_serror_rate, rerror_rate, srv_rerror_rate, same_srv_rate, diff_srv_rate, srv_diff_host_rate, dst_host_count, dst_host_srv_count, dst_host_same_srv_rate, dst_host_diff_srv_rate, dst_host_same_src_port_rate, dst_host_srv_diff_host_rate, dst_host_srv_serror_rate, dst_host_rerror_rate, dst_host_srv_rerror_rate.

R2L	Services, flag, hot, logged_in, is_guest_login, count, same_srv_rate, dst_host_count, dst_host_srv_count, dst_host_same_srv_rate, dst_host_diff_srv_rate, dst_host_same_src_port_rate, dst_host_srv_diff_host_rate.

U2R	Duration, protocol_type, service, flag, src_bytes, dst_bytes, hot, logged_in, num_compromised, root_shell, num_root, num_file_creations, num_shells, count, srv_count, same_srv_rate, dst_host_count, dst_host_srv_count, dst_host_same_srv_rate, dst_host_same_src_port_rate.

Normal	Protocol_type, service, flag, src_bytes, dst_bytes, logged_in, count, srv_count, same_srv_rate, srv_diff_host_rate, dst_host_count, dst_host_srv_count, dst_host_same_srv_rate, dst_host_same_src_port_rate.

**Table 4:** Tenfold cross validation results of DoS model.

Fold	*λ* = 1	*λ* = 1.5	*λ* = 2	*λ* = 2.5	*λ* = 3
1	100	100	100	100	100
2	99.36	99.40	99.40	99.44	99.44
3	99.80	99.86	99.88	99.88	99.92
4	100	100	100	100	100
5	99.76	99.78	99.82	99.84	99.88
6	100	100	100	100	100
7	99.04	99.04	99.04	99.08	99.10
8	80.16	84.70	85.74	85.82	85.90
9	99.42	99.46	99.52	99.52	99.54
10	99.84	99.92	99.92	99.92	99.92
Avg.	**97.59**	**98.22**	**98.22**	**98.35**	**98.37**

**Table 5:** Average detection rates (%) of different models with 10-fold cross validation technique.

Class	*μ*_TSD_ + 1*∗σ*_TSD_	*μ*_TSD_ + 1.5*∗σ*_TSD_	*μ*_TSD_ + 2*∗σ*_TSD_	*μ*_TSD_ + 2.5*∗σ*_TSD_	*μ*_TSD_ + 3*∗σ*_TSD_
Normal	97.34	98.16	98.97	99.60	99.76
DoS	97.59	98.22	98.22	98.35	98.37
Probe	91.55	94.15	95.48	96.44	98.07
R2L	89.50	96.00	96.25	97.50	98.25
U2R	45.00	50.00	60.00	60.00	60.00

**Table 6:** Testing performances for five classes.

Class	Evaluation metrics	*μ*_TSD_ + 1*∗σ*_TSD_	*μ*_TSD_ + 2*∗σ*_TSD_	*μ*_TSD_ + 3*∗σ*_TSD_
Normal	DR (%)	100	100	100
	FAR (%)	3.53	1.02	0.30

DoS	DR (%)	99.74	99.75	99.77
	FAR (%)	0.26	0.23	0.23

Probe	DR (%)	96.73	95.52	97.32
	FAR (%)	3.67	2.54	0.94

R2L	DR (%)	100	100	100
	FAR (%)	10.5	3.5	2.50

U2R	DR (%)	100	100	100
	FAR (%)	62	52	44

**Table 7:** Accuracy (%) achieved by the proposed system for different thresholds.

Threshold	Normal	DoS	Probe	R2l	U2R
*μ*_TSD_ + 1*∗σ*_TSD_	98.18	99.66	96.88	92.7	64
*μ*_TSD_ + 2*∗σ*_TSD_	99.49	99.36	72.27	98.25	69
*μ*_TSD_ + 3*∗σ*_TSD_	99.85	99.25	59.31	92.88	53

## References

[1] Luo B., Xia J. (2014). A novel intrusion detection system based on feature generation with visualization strategy. *Expert Systems with Applications*.

[2] Elhag S., Fernández A., Bawakid A., Alshomrani S., Herrera F. (2015). On the combination of genetic fuzzy systems and pairwise learning for improving detection rates on Intrusion Detection Systems. *Expert Systems with Applications*.

[3] Derhab A., Bouras A. (2015). Multivariate correlation analysis and geometric linear similarity for real-time intrusion detection systems. *Security and Communication Networks*.

[4] Tsai C.-F., Lin C.-Y. (2010). A triangle area based nearest neighbors approach to intrusion detection. *Pattern Recognition*.

[5] Chen T., Zhang X., Jin S., Kim O. (2014). Efficient classification using parallel and scalable compressed model and its application on intrusion detection. *Expert Systems with Applications*.

[6] Ganapathy S., Yogesh P., Kannan A. (2012). Intelligent agent-based intrusion detection system using enhanced multiclass SVM. *Computational Intelligence and Neuroscience*.

[7] Sindhu S. S. S., Geetha S., Kannan A. (2012). Decision tree based light weight intrusion detection using a wrapper approach. *Expert Systems with Applications*.

[8] Sarasamma S. T., Zhu Q. A., Huff J. (2005). Hierarchical Kohonenen Net for anomaly detection in network security. *IEEE Transactions on Systems, Man, and Cybernetics, Part B: Cybernetics*.

[9] Denning D. E. (1987). An intrusion-detection model. *IEEE Transactions on Software Engineering*.

[10] Lin S.-W., Ying K.-C., Lee C.-Y., Lee Z.-J. (2012). An intelligent algorithm with feature selection and decision rules applied to anomaly intrusion detection. *Applied Soft Computing Journal*.

[11] Goonatilake R., Herath S., Herath A. (2013). Probabilistic models for anomaly detection based on usage of network traffic. *Journal of Information Engineering and Applications*.

[12] Ye N., Li X., Chen Q., Emran S. M., Xu M. (2001). Probabilistic techniques for intrusion detection based on computer audit data. *IEEE Transactions on Systems, Man, and Cybernetics, Part A: Systems and Humans*.

[13] Ye N., Chen Q. (2001). An anomaly detection technique based on a chi-square statistic for detecting intrusions into information systems. *Quality and Reliability Engineering International*.

[14] Tan Z., Jamdagni A., He X., Nanda P., Liu R. P. (2014). A system for denial-of-service attack detection based on multivariate correlation analysis. *IEEE Transactions on Parallel and Distributed Systems*.

[15] Ye N., Emran S. M., Chen Q., Vilbert S. (2002). Multivariate statistical analysis of audit trails for host-based intrusion detection. *IEEE Transactions on Computers*.

[16] Talib M. A., Munisamy S., Ahmed S. (2014). Retrospective Hotelling's T2 control chart for automotive stamped parts: a case study. *Journal of Science and Technology*.

[17] Chou Y.-M., Mason R. L., Young J. C. (1999). Power comparisons for a hotelling's T^2^ statistic. *Communications in Statistics Part B: Simulation and Computation*.

[18] Lu Y., Liu P.-Y., Xiao P., Deng H.-W. (2005). Hotelling's T2 multivariate profiling for detecting differential expression in microarrays. *Bioinformatics*.

[19] Ye N., Chen Q., Borror C. M. (2004). EWMA forecast of normal system activity for computer intrusion detection. *IEEE Transactions on Reliability*.

[20] Acharya A. K., Sahoo B., Swain B. R. Object tracking using a new statistical multivariate Hotelling's T^2^ approach.

[21] Sparks R. (2014). Monitoring highly correlated multivariate processes using hotelling's T2 statistic: problems and possible solutions. *Quality and Reliability Engineering International*.

[22] http://kdd.ics.uci.edu/databases/kddcup99/kddcup99.html.

[23] Han J., Kamber M., Pei J. (2006). *Data Mining, Southeast Asia Edition: Concepts and Techniques*.

